# Crystal structure of 4-*tert*-butyl-2-{2-[*N*-(3,3-dimethyl-2-oxobut­yl)-*N*-iso­propyl­carbamo­yl]phen­yl}-1-isopropyl-1*H*-imidazol-3-ium perchlorate

**DOI:** 10.1107/S2056989015001486

**Published:** 2015-01-28

**Authors:** Olga V. Hordiyenko, Roman I. Zubatyuk

**Affiliations:** a Taras Shevchenko National University of Kyiv, Department of Chemistry, 64/13 Volodymyrska str., Kyiv 01601, Ukraine; b SSI Institute for Single Crystals NAS of Ukraine, 60 Lenin ave., Kharkiv 61001, Ukraine

**Keywords:** crystal structure, α-acyl­amino ketone, perchlorate, disorder

## Abstract

In the title salt, C_26_H_40_N_3_O_2_^+^·ClO_4_^−^, the positive charge of the organic cation is delocalized between the two N atoms of the imidazole ring. The C N bond distances are 1.338 (2) and 1.327 (3) Å. The substituents on the benzene ring are rotated almost orthogonal with respect to this ring due to the presence of the bulky isopropyl substituents. The dihedral angle between the benzene and imidazole rings is 75.15 (12)°. Three of the O atoms of the anion are disordered over two sets of sites due to rotation around one of the O—Cl bonds. The ratio of the refined occupancies is 0.591 (14):0.409 (14). In the crystal, the cation and perchlorate anion are bound by an N—H⋯O hydrogen bond. In addition, the cation–anion pairs are linked into layers parallel to (001) by multiple weak C—H⋯O hydrogen bonds.

## Abstract 2

Bulky isopropyl substituents introduce steric hindrance within the mol­ecule. The organic cation and perchlorate anion are linked by N—H⋯O hydrogen bonding. In the crystal, mol­ecules form separated layers resulting in a low crystal density of 1.18 Mg m^−1^.

## Chemical context   

α-Amino­ketones are known for their fungicidal activity (Gold de Sigman, 1983 ▸) and 2-acyl­amino­ketones are the starting compounds in the oxazole synthetic method by the Robinson–Gabriel synthesis by dehydration of 2-acyl­amino­ketones (Robinson, 1909 ▸; Gabriel, 1910 ▸; Wasserman & Vinick, 1973 ▸) that has been used during studies dealing with pharmaceut­ically important mol­ecules that incorporate an oxazole deriv­ative (Godfrey *et al.*, 2003 ▸; Nicolaou *et al.*, 2004 ▸; Hoffman *et al.*, 2010 ▸).
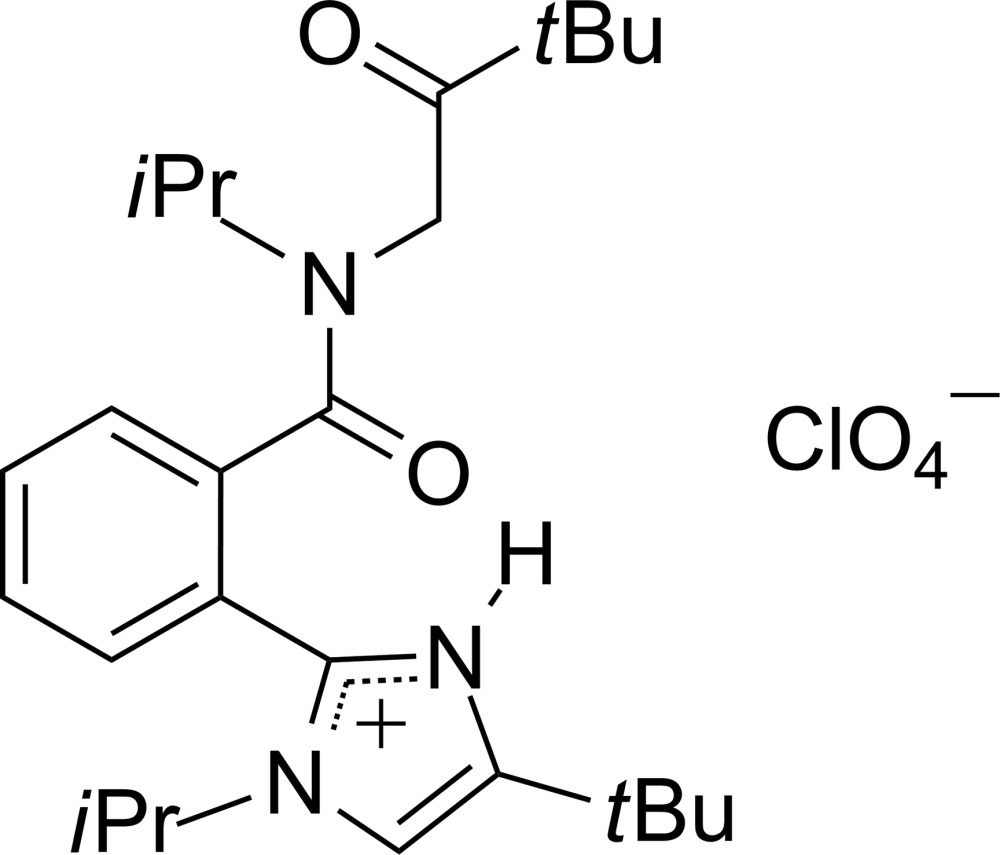


## Structural commentary   

**Figure 1:**
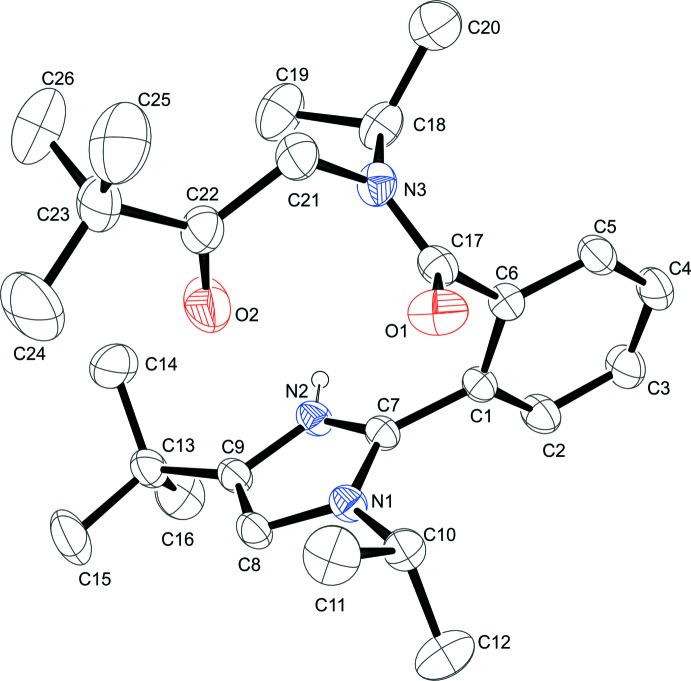
View of the title compound showing the atom-numbering scheme and 30% probability displacement ellipsoids. For clarity, the ClO_4_^−^ anion and H atoms are not shown.

The mol­ecular structure of the cation is shown in Fig. 1 ▸. The positive charge is delocalized between the two nitro­gen atoms of the imidazole ring according to almost equivalent lengths of the C7—N1 and C7—N2 bonds [1.338 (2) Å and 1.327 (3) Å, respectively] and also of the C8—N1 and C9—N2 bonds [1.379 (3) Å and 1.374 (3) Å, respectively]. The presence of two bulky substituents in the *ortho* positions of the benzene ring results in disruption of the conjugation between the aromatic ring, imidazole ring and amide [N3/C17/O1] fragment due to their almost orthogonal orientation [the corresponding torsion angles are N1—C7—C1—C6 = −81.5 (3)° and C5—C6—C17—N3 = 81.1 (3)°]. The plane of the carbonyl group (C22/O2/C23/C21) is oriented almost orthogonal to the plane of the amide fragment (C21/N3/C18/C17/O1/C6), the angle between their mean planes being 77.87 (11)°. A similar type of α-acyl­amino­ketone fragment has been observed for other *N*-substituted α-acyl­amino­ketones (Bartnik *et al.*, 1998 ▸; Tinant *et al.*, 2006 ▸; Chai *et al.*, 2011 ▸; Hashmi *et al.*, 2011 ▸; Su *et al.*, 2011 ▸).

The organic cation and perchlorate anion are linked by an N—H⋯O hydrogen bond (Table 1 ▸). The oxygen atoms of the anion are disordered over two sets of sites due to rotation around the O3—Cl bond. The refined occupancy of the major disordered component is 0.591 (14).

## Supra­molecular features   

**Figure 2:**
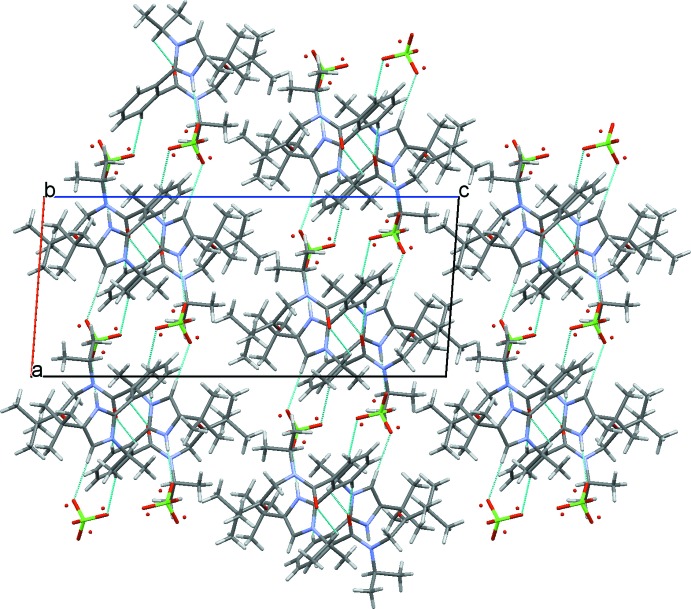
Part of the crystal structure, viewed along the *b* axis, showing layers parallel to (001) formed by weak C—H⋯O hydrogen bonds (turquoise dotted lines) and also separated layers of organic cations parallel to (101). The minor disorder component of the anion is shown as red spheres.

Several moderate to weak C—H⋯O inter­molecular hydrogen bonds are observed in the crystal structure (Table 1 ▸), which link mol­ecules into layers parallel to (001) (Fig. 2 ▸). It should also be noted that the crystal structure contains no residual solvent-accessible voids. However, discernible layers along (101) are observed. The low density [1.18 g mm^−1^] of the crystal could be associated with formation of these layers.

## Synthesis and crystallization   

The title compound was synthesized according to the literature procedure (Hordiyenko *et al.*, 2009 ▸). To a stirred solution of 1-(*N*-iso­propyl­amino)-3,3-di­methyl­butan-2-one (10 mmol) in dry CHCl_3_ (10 mL), a solution of 1,1,3-tri­chloro-1*H*-iso­indole (2.5 mmol) in dry CHCl_3_ (10 mL) was added dropwise at room temperature under an argon atmosphere. The reaction mixture was stirred for 8 h, the solvent was evaporated and the residue was dried under reduced pressure (0.01 mm). Then it was treated with 100 ml of distilled water. The aqueous solution was brought to reflux with charcoal, filtered and treated with an excess of lithium perchlorate to precipitate the crude product that was then crystallized from methanol/water (3:1) to yield as colorless crystals. Single crystals suitable for X-ray diffraction were obtained by slow evaporation of a solution of the title compound in ethanol.

## Refinement   

Crystal data, data collection and structure refinement details are summarized in Table 2 ▸. Hydrogen atoms were placed in calculated positions (N—H = 0.86 Å, C—H = 0.93–0.98 Å) and refined in a riding-model approximation with *U*_iso_ = *nU*_eq_ of the carrier atom (*n* = 1.5 for methyl groups, *n* = 1.2 for the remaining H atoms). Methyl groups were refined as rotating groups. The relative occupation of the two positions of the disordered ClO_4_ anion was refined as a free variable. All Cl—O and O⋯O distances within the anion were restrained to be the same within 0.02 Å.

## Supplementary Material

Crystal structure: contains datablock(s) I. DOI: 10.1107/S2056989015001486/lh5748sup1.cif

Structure factors: contains datablock(s) I. DOI: 10.1107/S2056989015001486/lh5748Isup2.hkl

Supporting information file. DOI: 10.1107/S2056989015001486/lh5748Isup3.smi

Supporting information file. DOI: 10.1107/S2056989015001486/lh5748Isup4.cml

CCDC reference: 1045018

Additional supporting information:  crystallographic information; 3D view; checkCIF report

## Figures and Tables

**Table 1:** Hydrogen-bond geometry (, )

*D*H*A*	*D*H	H*A*	*D* *A*	*D*H*A*
N2H2O3	0.86	1.94	2.752(4)	157
C2H2*A*O1^i^	0.93	2.44	3.319(3)	158
C5H5O5*A* ^ii^	0.93	2.55	3.328(11)	141
C8H8O4*A* ^iii^	0.93	2.36	3.285(8)	173

Symmetry codes: (i) 
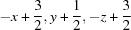
; (ii) 
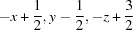
; (iii) 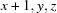.

**Table 2:** Experimental details

Crystal data	
Chemical formula	C_26_H_40_N_3_O_2_^+^ClO_4_
*M*_r_	526.06
Crystal system, space group	Monoclinic, *P*2_1_/*n*
Temperature (K)	293
*a*, *b*, *c* ()	10.0605(3), 12.7027(4), 23.1455(6)
()	94.107(3)
*V* (^3^)	2950.29(14)
*Z*	4
Radiation type	Mo *K*
(mm^1^)	0.17
Crystal size (mm)	0.57 0.32 0.09

Data collection	
Diffractometer	Agilent Xcalibur Sapphire3
Absorption correction	Multi-scan (*CrysAlis PRO*; Agilent, 2014 ▸)
*T*_min_, *T*_max_	0.951, 1.000
No. of measured, independent and observed [*I* > 2(*I*)] reflections	27330, 6037, 4458
*R*_int_	0.029
(sin /)_max_ (^1^)	0.626

Refinement	
*R*[*F* ^2^ > 2(*F* ^2^)], *wR*(*F* ^2^), *S*	0.060, 0.179, 1.04
No. of reflections	6037
No. of parameters	363
No. of restraints	87
H-atom treatment	H-atom parameters constrained
_max_, _min_ (e ^3^)	0.37, 0.33

Computer programs: *CrysAlis PRO* (Agilent, 2014 ▸), *SHELXD* (Sheldrick, 2008 ▸), *SHELXL97* (Sheldrick, 2015 ▸) and *OLEX2* (Dolomanov *et al.*, 2009 ▸).

## supplementary crystallographic information

### Crystal data

**Table:**

C_26_H_40_N_3_O_2_^+^·ClO_4_^−^	*F*(000) = 1128
*M**_r_* = 526.06	*D*_x_ = 1.184 Mg m^−^^3^
Monoclinic, *P*2_1_/*n*	Mo *K*α radiation, λ = 0.71073 Å
*a* = 10.0605 (3) Å	Cell parameters from 6208 reflections
*b* = 12.7027 (4) Å	θ = 3.2–26.2°
*c* = 23.1455 (6) Å	µ = 0.17 mm^−^^1^
β = 94.107 (3)°	*T* = 293 K
*V* = 2950.29 (14) Å^3^	Block, colorless
*Z* = 4	0.57 × 0.32 × 0.09 mm

### Data collection

**Table:**

Agilent Xcalibur Sapphire3 diffractometer	6037 independent reflections
Radiation source: Enhance (Mo) X-ray Source	4458 reflections with *I* > 2σ(*I*)
Graphite monochromator	*R*_int_ = 0.029
Detector resolution: 16.1827 pixels mm^-1^	θ_max_ = 26.4°, θ_min_ = 3.1°
ω and π scans	*h* = −12→12
Absorption correction: multi-scan (*CrysAlis PRO*; Agilent, 2014)	*k* = −15→15
*T*_min_ = 0.951, *T*_max_ = 1.000	*l* = −28→28
27330 measured reflections	

### Refinement

**Table:**

Refinement on *F*^2^	Primary atom site location: dual
Least-squares matrix: full	Hydrogen site location: inferred from neighbouring sites
*R*[*F*^2^ > 2σ(*F*^2^)] = 0.060	H-atom parameters constrained
*wR*(*F*^2^) = 0.179	*w* = 1/[σ^2^(*F*_o_^2^) + (0.0837*P*)^2^ + 1.4781*P*] where *P* = (*F*_o_^2^ + 2*F*_c_^2^)/3
*S* = 1.04	(Δ/σ)_max_ < 0.001
6037 reflections	Δρ_max_ = 0.37 e Å^−^^3^
363 parameters	Δρ_min_ = −0.33 e Å^−^^3^
87 restraints	

### Special details

**Table:**

Geometry. All e.s.d.'s (except the e.s.d. in the dihedral angle between two l.s. planes) are estimated using the full covariance matrix. The cell e.s.d.'s are taken into account individually in the estimation of e.s.d.'s in distances, angles and torsion angles; correlations between e.s.d.'s in cell parameters are only used when they are defined by crystal symmetry. An approximate (isotropic) treatment of cell e.s.d.'s is used for estimating e.s.d.'s involving l.s. planes.

### Fractional atomic coordinates and isotropic or equivalent isotropic displacement parameters (Å^2^)

**Table:**

	*x*	*y*	*z*	*U*_iso_*/*U*_eq_	Occ. (<1)
O1	0.72746 (17)	0.14319 (15)	0.71062 (9)	0.0685 (5)	
O2	0.7339 (3)	0.25979 (17)	0.58916 (9)	0.0915 (7)	
N2	0.67413 (16)	0.47485 (14)	0.66538 (8)	0.0421 (4)	
H2	0.5935	0.4962	0.6583	0.051*	
N1	0.84449 (16)	0.39425 (14)	0.70494 (7)	0.0416 (4)	
C7	0.71299 (19)	0.40741 (15)	0.70699 (9)	0.0383 (4)	
C8	0.8863 (2)	0.45571 (18)	0.66063 (9)	0.0450 (5)	
H8	0.9735	0.4614	0.6501	0.054*	
C1	0.62173 (19)	0.36474 (16)	0.74861 (9)	0.0398 (4)	
C6	0.5700 (2)	0.26255 (16)	0.74384 (9)	0.0420 (5)	
C9	0.7801 (2)	0.50599 (18)	0.63519 (9)	0.0442 (5)	
N3	0.54488 (19)	0.17087 (15)	0.65053 (8)	0.0496 (5)	
C17	0.6201 (2)	0.18761 (16)	0.70018 (10)	0.0463 (5)	
C10	0.9309 (2)	0.32719 (18)	0.74425 (10)	0.0488 (5)	
H10	0.8739	0.2799	0.7650	0.059*	
C2	0.5770 (2)	0.43279 (19)	0.79004 (11)	0.0537 (6)	
H2A	0.6106	0.5009	0.7931	0.064*	
C5	0.4771 (2)	0.23065 (19)	0.78176 (10)	0.0538 (6)	
H5	0.4437	0.1624	0.7795	0.065*	
C4	0.4338 (3)	0.2986 (2)	0.82254 (11)	0.0609 (7)	
H4	0.3710	0.2763	0.8475	0.073*	
C21	0.6048 (3)	0.10861 (19)	0.60668 (11)	0.0570 (6)	
H21A	0.5351	0.0816	0.5795	0.068*	
H21B	0.6509	0.0490	0.6250	0.068*	
C13	0.7626 (2)	0.5776 (2)	0.58316 (11)	0.0566 (6)	
C3	0.4831 (3)	0.3996 (2)	0.82658 (11)	0.0625 (7)	
H3	0.4529	0.4456	0.8540	0.075*	
C22	0.7029 (3)	0.1726 (2)	0.57379 (11)	0.0625 (7)	
C18	0.4094 (3)	0.2141 (2)	0.63707 (11)	0.0592 (6)	
H18	0.3952	0.2695	0.6654	0.071*	
C11	1.0206 (3)	0.2608 (3)	0.70900 (14)	0.0773 (8)	
H11A	0.9682	0.2274	0.6779	0.116*	
H11B	1.0637	0.2080	0.7334	0.116*	
H11C	1.0867	0.3049	0.6934	0.116*	
C12	1.0089 (3)	0.3951 (3)	0.78810 (14)	0.0817 (9)	
H12A	1.0651	0.4422	0.7685	0.123*	
H12B	1.0630	0.3512	0.8141	0.123*	
H12C	0.9484	0.4351	0.8097	0.123*	
C14	0.6630 (4)	0.5257 (3)	0.53881 (14)	0.0929 (11)	
H14A	0.6956	0.4577	0.5286	0.139*	
H14B	0.6524	0.5689	0.5048	0.139*	
H14C	0.5786	0.5181	0.5552	0.139*	
C20	0.3049 (3)	0.1288 (3)	0.64423 (14)	0.0758 (8)	
H20A	0.3203	0.0711	0.6188	0.114*	
H20B	0.2177	0.1573	0.6348	0.114*	
H20C	0.3111	0.1045	0.6836	0.114*	
C16	0.7086 (4)	0.6841 (3)	0.60196 (16)	0.0877 (10)	
H16A	0.6257	0.6736	0.6193	0.132*	
H16B	0.6946	0.7291	0.5688	0.132*	
H16C	0.7717	0.7162	0.6296	0.132*	
C23	0.7609 (3)	0.1226 (3)	0.52116 (13)	0.0782 (9)	
C25	0.7743 (5)	0.0024 (3)	0.52725 (19)	0.1192 (15)	
H25A	0.8327	−0.0139	0.5607	0.179*	
H25B	0.8104	−0.0259	0.4933	0.179*	
H25C	0.6882	−0.0278	0.5316	0.179*	
C15	0.8972 (3)	0.5924 (3)	0.55791 (15)	0.0943 (11)	
H15A	0.9590	0.6232	0.5866	0.141*	
H15B	0.8870	0.6379	0.5248	0.141*	
H15C	0.9304	0.5253	0.5464	0.141*	
C19	0.3955 (4)	0.2640 (3)	0.57734 (13)	0.0843 (9)	
H19A	0.4649	0.3151	0.5741	0.127*	
H19B	0.3103	0.2980	0.5718	0.127*	
H19C	0.4024	0.2106	0.5484	0.127*	
C26	0.6614 (5)	0.1441 (4)	0.46941 (16)	0.1277 (16)	
H26A	0.5769	0.1131	0.4764	0.192*	
H26B	0.6939	0.1139	0.4351	0.192*	
H26C	0.6508	0.2187	0.4643	0.192*	
C24	0.8914 (5)	0.1763 (5)	0.5105 (3)	0.177 (3)	
H24A	0.8760	0.2501	0.5039	0.265*	
H24B	0.9277	0.1458	0.4771	0.265*	
H24C	0.9532	0.1671	0.5436	0.265*	
Cl1	0.29860 (6)	0.54707 (6)	0.65153 (4)	0.0755 (3)	
O3	0.4025 (3)	0.4841 (3)	0.64132 (19)	0.1626 (16)	
O4A	0.1909 (7)	0.4970 (13)	0.6233 (5)	0.233 (7)	0.591 (14)
O5A	0.2680 (11)	0.5578 (8)	0.7063 (3)	0.177 (5)	0.591 (14)
O6A	0.3130 (16)	0.6413 (6)	0.6273 (7)	0.276 (10)	0.591 (14)
O6B	0.3652 (14)	0.6383 (9)	0.6691 (8)	0.235 (10)	0.409 (14)
O4B	0.2175 (13)	0.5692 (15)	0.6066 (6)	0.216 (10)	0.409 (14)
O5B	0.2331 (17)	0.5113 (17)	0.6954 (8)	0.316 (17)	0.409 (14)

### Atomic displacement parameters (Å^2^)

**Table:**

	*U*^11^	*U*^22^	*U*^33^	*U*^12^	*U*^13^	*U*^23^
O1	0.0517 (10)	0.0604 (11)	0.0927 (13)	0.0105 (8)	0.0006 (9)	−0.0208 (10)
O2	0.1285 (19)	0.0672 (13)	0.0840 (14)	−0.0322 (13)	0.0444 (13)	−0.0187 (11)
N2	0.0307 (8)	0.0455 (10)	0.0506 (10)	0.0019 (7)	0.0051 (7)	0.0055 (8)
N1	0.0334 (8)	0.0437 (9)	0.0480 (10)	0.0036 (7)	0.0054 (7)	0.0018 (8)
C7	0.0356 (10)	0.0363 (10)	0.0435 (10)	−0.0001 (8)	0.0072 (8)	−0.0008 (8)
C8	0.0339 (10)	0.0532 (12)	0.0489 (12)	−0.0025 (9)	0.0100 (8)	0.0042 (10)
C1	0.0357 (10)	0.0408 (11)	0.0437 (11)	0.0003 (8)	0.0075 (8)	0.0001 (9)
C6	0.0419 (11)	0.0407 (11)	0.0437 (11)	−0.0004 (8)	0.0062 (8)	0.0012 (9)
C9	0.0364 (10)	0.0496 (12)	0.0471 (11)	−0.0044 (9)	0.0064 (8)	0.0047 (9)
N3	0.0583 (11)	0.0440 (10)	0.0474 (10)	0.0052 (8)	0.0108 (8)	−0.0045 (8)
C17	0.0500 (12)	0.0341 (10)	0.0562 (13)	−0.0023 (9)	0.0129 (10)	−0.0007 (9)
C10	0.0425 (11)	0.0502 (13)	0.0532 (12)	0.0078 (9)	0.0003 (9)	0.0068 (10)
C2	0.0567 (14)	0.0449 (12)	0.0614 (14)	−0.0075 (10)	0.0185 (11)	−0.0092 (10)
C5	0.0567 (14)	0.0501 (13)	0.0561 (13)	−0.0130 (10)	0.0151 (11)	0.0002 (10)
C4	0.0593 (14)	0.0713 (17)	0.0550 (14)	−0.0132 (12)	0.0241 (11)	−0.0034 (12)
C21	0.0699 (16)	0.0465 (13)	0.0565 (14)	0.0007 (11)	0.0176 (12)	−0.0113 (11)
C13	0.0482 (13)	0.0664 (15)	0.0549 (13)	−0.0077 (11)	0.0015 (10)	0.0206 (12)
C3	0.0661 (16)	0.0669 (16)	0.0578 (14)	−0.0076 (13)	0.0273 (12)	−0.0159 (12)
C22	0.0750 (17)	0.0586 (16)	0.0553 (14)	−0.0039 (13)	0.0150 (12)	−0.0095 (12)
C18	0.0714 (16)	0.0542 (14)	0.0512 (13)	0.0172 (12)	−0.0007 (11)	−0.0072 (11)
C11	0.0663 (17)	0.0764 (19)	0.090 (2)	0.0317 (15)	0.0076 (15)	−0.0001 (16)
C12	0.085 (2)	0.079 (2)	0.0765 (19)	0.0132 (17)	−0.0260 (16)	−0.0101 (16)
C14	0.104 (3)	0.107 (3)	0.0633 (18)	−0.032 (2)	−0.0244 (17)	0.0306 (18)
C20	0.0575 (16)	0.087 (2)	0.083 (2)	0.0093 (14)	0.0054 (14)	−0.0102 (16)
C16	0.093 (2)	0.071 (2)	0.099 (2)	0.0079 (17)	0.0018 (18)	0.0326 (18)
C23	0.084 (2)	0.088 (2)	0.0668 (17)	−0.0032 (16)	0.0307 (15)	−0.0167 (15)
C25	0.150 (4)	0.109 (3)	0.103 (3)	0.036 (3)	0.042 (3)	−0.029 (2)
C15	0.0691 (19)	0.134 (3)	0.082 (2)	−0.0079 (19)	0.0244 (16)	0.048 (2)
C19	0.115 (3)	0.076 (2)	0.0605 (17)	0.0214 (18)	−0.0077 (16)	0.0058 (15)
C26	0.169 (4)	0.150 (4)	0.064 (2)	0.021 (3)	0.011 (2)	−0.016 (2)
C24	0.136 (4)	0.234 (7)	0.174 (5)	−0.073 (4)	0.105 (4)	−0.088 (5)
Cl1	0.0441 (4)	0.0817 (5)	0.1017 (6)	0.0188 (3)	0.0118 (3)	−0.0090 (4)
O3	0.0613 (15)	0.151 (3)	0.274 (5)	0.0386 (17)	0.002 (2)	−0.070 (3)
O4A	0.054 (3)	0.45 (2)	0.195 (10)	−0.068 (7)	0.005 (4)	−0.011 (11)
O5A	0.236 (12)	0.177 (8)	0.126 (6)	0.064 (8)	0.076 (6)	−0.025 (5)
O6A	0.38 (2)	0.108 (6)	0.37 (2)	0.114 (9)	0.229 (15)	0.112 (9)
O6B	0.272 (16)	0.184 (13)	0.248 (18)	−0.150 (12)	0.012 (13)	−0.095 (12)
O4B	0.089 (9)	0.35 (3)	0.194 (12)	0.039 (11)	−0.079 (9)	0.058 (14)
O5B	0.179 (14)	0.45 (3)	0.33 (3)	−0.035 (18)	0.145 (18)	0.20 (3)

### Geometric parameters (Å, º)

**Table:**

O1—C17	1.227 (3)	C11—H11B	0.9600
O2—C22	1.197 (3)	C11—H11C	0.9600
N2—H2	0.8600	C12—H12A	0.9600
N2—C7	1.327 (3)	C12—H12B	0.9600
N2—C9	1.374 (3)	C12—H12C	0.9600
N1—C7	1.338 (2)	C14—H14A	0.9600
N1—C8	1.379 (3)	C14—H14B	0.9600
N1—C10	1.482 (3)	C14—H14C	0.9600
C7—C1	1.480 (3)	C20—H20A	0.9600
C8—H8	0.9300	C20—H20B	0.9600
C8—C9	1.344 (3)	C20—H20C	0.9600
C1—C6	1.400 (3)	C16—H16A	0.9600
C1—C2	1.389 (3)	C16—H16B	0.9600
C6—C17	1.501 (3)	C16—H16C	0.9600
C6—C5	1.388 (3)	C23—C25	1.537 (5)
C9—C13	1.510 (3)	C23—C26	1.530 (5)
N3—C17	1.347 (3)	C23—C24	1.516 (5)
N3—C21	1.451 (3)	C25—H25A	0.9600
N3—C18	1.482 (3)	C25—H25B	0.9600
C10—H10	0.9800	C25—H25C	0.9600
C10—C11	1.515 (4)	C15—H15A	0.9600
C10—C12	1.508 (4)	C15—H15B	0.9600
C2—H2A	0.9300	C15—H15C	0.9600
C2—C3	1.379 (3)	C19—H19A	0.9600
C5—H5	0.9300	C19—H19B	0.9600
C5—C4	1.373 (3)	C19—H19C	0.9600
C4—H4	0.9300	C26—H26A	0.9600
C4—C3	1.376 (4)	C26—H26B	0.9600
C21—H21A	0.9700	C26—H26C	0.9600
C21—H21B	0.9700	C24—H24A	0.9600
C21—C22	1.524 (4)	C24—H24B	0.9600
C13—C14	1.532 (4)	C24—H24C	0.9600
C13—C16	1.532 (4)	Cl1—O3	1.350 (3)
C13—C15	1.524 (4)	Cl1—O4A	1.380 (7)
C3—H3	0.9300	Cl1—O5A	1.333 (6)
C22—C23	1.526 (4)	Cl1—O6A	1.334 (6)
C18—H18	0.9800	Cl1—O6B	1.385 (7)
C18—C20	1.527 (4)	Cl1—O4B	1.306 (8)
C18—C19	1.518 (4)	Cl1—O5B	1.329 (9)
C11—H11A	0.9600		
			
C7—N2—H2	124.6	C10—C12—H12A	109.5
C7—N2—C9	110.85 (17)	C10—C12—H12B	109.5
C9—N2—H2	124.6	C10—C12—H12C	109.5
C7—N1—C8	108.12 (17)	H12A—C12—H12B	109.5
C7—N1—C10	126.04 (18)	H12A—C12—H12C	109.5
C8—N1—C10	125.83 (17)	H12B—C12—H12C	109.5
N2—C7—N1	107.20 (17)	C13—C14—H14A	109.5
N2—C7—C1	122.99 (17)	C13—C14—H14B	109.5
N1—C7—C1	129.68 (18)	C13—C14—H14C	109.5
N1—C8—H8	125.7	H14A—C14—H14B	109.5
C9—C8—N1	108.64 (18)	H14A—C14—H14C	109.5
C9—C8—H8	125.7	H14B—C14—H14C	109.5
C6—C1—C7	122.16 (18)	C18—C20—H20A	109.5
C2—C1—C7	117.89 (19)	C18—C20—H20B	109.5
C2—C1—C6	119.65 (19)	C18—C20—H20C	109.5
C1—C6—C17	120.04 (18)	H20A—C20—H20B	109.5
C5—C6—C1	119.0 (2)	H20A—C20—H20C	109.5
C5—C6—C17	120.9 (2)	H20B—C20—H20C	109.5
N2—C9—C13	121.92 (19)	C13—C16—H16A	109.5
C8—C9—N2	105.19 (18)	C13—C16—H16B	109.5
C8—C9—C13	132.8 (2)	C13—C16—H16C	109.5
C17—N3—C21	116.5 (2)	H16A—C16—H16B	109.5
C17—N3—C18	124.95 (18)	H16A—C16—H16C	109.5
C21—N3—C18	118.49 (19)	H16B—C16—H16C	109.5
O1—C17—C6	119.5 (2)	C22—C23—C25	112.1 (3)
O1—C17—N3	121.9 (2)	C22—C23—C26	106.6 (3)
N3—C17—C6	118.65 (19)	C26—C23—C25	107.3 (3)
N1—C10—H10	108.4	C24—C23—C22	109.1 (3)
N1—C10—C11	109.6 (2)	C24—C23—C25	113.0 (4)
N1—C10—C12	109.8 (2)	C24—C23—C26	108.5 (4)
C11—C10—H10	108.4	C23—C25—H25A	109.5
C12—C10—H10	108.4	C23—C25—H25B	109.5
C12—C10—C11	112.3 (2)	C23—C25—H25C	109.5
C1—C2—H2A	119.9	H25A—C25—H25B	109.5
C3—C2—C1	120.2 (2)	H25A—C25—H25C	109.5
C3—C2—H2A	119.9	H25B—C25—H25C	109.5
C6—C5—H5	119.6	C13—C15—H15A	109.5
C4—C5—C6	120.8 (2)	C13—C15—H15B	109.5
C4—C5—H5	119.6	C13—C15—H15C	109.5
C5—C4—H4	119.9	H15A—C15—H15B	109.5
C5—C4—C3	120.2 (2)	H15A—C15—H15C	109.5
C3—C4—H4	119.9	H15B—C15—H15C	109.5
N3—C21—H21A	109.2	C18—C19—H19A	109.5
N3—C21—H21B	109.2	C18—C19—H19B	109.5
N3—C21—C22	112.0 (2)	C18—C19—H19C	109.5
H21A—C21—H21B	107.9	H19A—C19—H19B	109.5
C22—C21—H21A	109.2	H19A—C19—H19C	109.5
C22—C21—H21B	109.2	H19B—C19—H19C	109.5
C9—C13—C14	108.0 (2)	C23—C26—H26A	109.5
C9—C13—C16	109.2 (2)	C23—C26—H26B	109.5
C9—C13—C15	108.9 (2)	C23—C26—H26C	109.5
C14—C13—C16	110.0 (3)	H26A—C26—H26B	109.5
C15—C13—C14	110.6 (3)	H26A—C26—H26C	109.5
C15—C13—C16	110.1 (3)	H26B—C26—H26C	109.5
C2—C3—H3	119.9	C23—C24—H24A	109.5
C4—C3—C2	120.2 (2)	C23—C24—H24B	109.5
C4—C3—H3	119.9	C23—C24—H24C	109.5
O2—C22—C21	120.6 (2)	H24A—C24—H24B	109.5
O2—C22—C23	121.1 (3)	H24A—C24—H24C	109.5
C21—C22—C23	118.3 (2)	H24B—C24—H24C	109.5
N3—C18—H18	107.6	O3—Cl1—O4A	103.6 (6)
N3—C18—C20	110.1 (2)	O3—Cl1—O6B	100.5 (6)
N3—C18—C19	111.7 (2)	O5A—Cl1—O3	117.5 (5)
C20—C18—H18	107.6	O5A—Cl1—O4A	105.7 (6)
C19—C18—H18	107.6	O5A—Cl1—O6A	110.3 (6)
C19—C18—C20	112.1 (2)	O6A—Cl1—O3	110.4 (5)
C10—C11—H11A	109.5	O6A—Cl1—O4A	108.7 (7)
C10—C11—H11B	109.5	O4B—Cl1—O3	115.8 (7)
C10—C11—H11C	109.5	O4B—Cl1—O6B	108.4 (8)
H11A—C11—H11B	109.5	O4B—Cl1—O5B	111.5 (8)
H11A—C11—H11C	109.5	O5B—Cl1—O3	111.3 (8)
H11B—C11—H11C	109.5	O5B—Cl1—O6B	108.6 (9)
			
O2—C22—C23—C25	148.6 (4)	C1—C2—C3—C4	0.5 (4)
O2—C22—C23—C26	−94.3 (4)	C6—C1—C2—C3	0.5 (4)
O2—C22—C23—C24	22.6 (5)	C6—C5—C4—C3	−0.4 (4)
N2—C7—C1—C6	103.3 (2)	C9—N2—C7—N1	0.5 (2)
N2—C7—C1—C2	−70.4 (3)	C9—N2—C7—C1	176.64 (19)
N2—C9—C13—C14	−56.9 (3)	N3—C21—C22—O2	8.0 (4)
N2—C9—C13—C16	62.6 (3)	N3—C21—C22—C23	−172.3 (2)
N2—C9—C13—C15	−177.1 (3)	C17—C6—C5—C4	178.5 (2)
N1—C7—C1—C6	−81.5 (3)	C17—N3—C21—C22	−78.2 (3)
N1—C7—C1—C2	104.8 (3)	C17—N3—C18—C20	−102.9 (3)
N1—C8—C9—N2	0.8 (2)	C17—N3—C18—C19	131.9 (2)
N1—C8—C9—C13	−175.9 (2)	C10—N1—C7—N2	178.70 (19)
C7—N2—C9—C8	−0.8 (2)	C10—N1—C7—C1	2.9 (3)
C7—N2—C9—C13	176.3 (2)	C10—N1—C8—C9	−179.2 (2)
C7—N1—C8—C9	−0.5 (2)	C2—C1—C6—C17	−178.5 (2)
C7—N1—C10—C11	133.0 (2)	C2—C1—C6—C5	−1.5 (3)
C7—N1—C10—C12	−103.2 (3)	C5—C6—C17—O1	−98.6 (3)
C7—C1—C6—C17	7.9 (3)	C5—C6—C17—N3	81.1 (3)
C7—C1—C6—C5	−175.1 (2)	C5—C4—C3—C2	−0.6 (4)
C7—C1—C2—C3	174.4 (2)	C21—N3—C17—O1	−7.9 (3)
C8—N1—C7—N2	0.0 (2)	C21—N3—C17—C6	172.35 (19)
C8—N1—C7—C1	−175.8 (2)	C21—N3—C18—C20	79.4 (3)
C8—N1—C10—C11	−48.5 (3)	C21—N3—C18—C19	−45.8 (3)
C8—N1—C10—C12	75.3 (3)	C21—C22—C23—C25	−31.2 (4)
C8—C9—C13—C14	119.3 (3)	C21—C22—C23—C26	85.9 (4)
C8—C9—C13—C16	−121.2 (3)	C21—C22—C23—C24	−157.1 (4)
C8—C9—C13—C15	−0.9 (4)	C18—N3—C17—O1	174.4 (2)
C1—C6—C17—O1	78.3 (3)	C18—N3—C17—C6	−5.4 (3)
C1—C6—C17—N3	−101.9 (2)	C18—N3—C21—C22	99.7 (3)
C1—C6—C5—C4	1.5 (4)		

### Hydrogen-bond geometry (Å, º)

**Table:**

*D*—H···*A*	*D*—H	H···*A*	*D*···*A*	*D*—H···*A*
N2—H2···O3	0.86	1.94	2.752 (4)	157
C2—H2*A*···O1^i^	0.93	2.44	3.319 (3)	158
C5—H5···O5*A*^ii^	0.93	2.55	3.328 (11)	141
C8—H8···O4*A*^iii^	0.93	2.36	3.285 (8)	173
C10—H10···O1	0.98	2.55	3.167 (3)	121

Symmetry codes: (i) −*x*+3/2, *y*+1/2, −*z*+3/2; (ii) −*x*+1/2, *y*−1/2, −*z*+3/2; (iii) *x*+1, *y*, *z*.
